# Case Report: Sustained immune and pulmonary recovery three years after hematopoietic stem cell transplantation for ITCH E3 ubiquitin ligase deficiency

**DOI:** 10.3389/fimmu.2026.1805196

**Published:** 2026-05-01

**Authors:** Brittany Ashe, Emily Neal, Geoffrey Kurland, Hey Chong, David Lacomis, Dana Olup, Emily Olup, Lina Ghaloul-Gonzalez, Jeffrey Rudolph, Nicole Hogue, Scott H. Maurer, Basil Zitelli, Paul Szabolcs, Kathryn S. Torok

**Affiliations:** 1 Division of Pediatric Rheumatology, Department of Pediatrics, University of Pittsburgh Medical Center (UPMC) Children’s Hospital of Pittsburgh, Pittsburgh, PA, United States; 2 University of Pittsburgh School of Medicine, Pittsburgh, PA, United States; 3 Division of Pediatric Pulmonology, Department of Pediatrics, UPMC Children’s Hospital of Pittsburgh, Pittsburgh, PA, United States; 4 Division of Allergy and Immunology, Department of Pediatrics, UPMC Children’s Hospital of Pittsburgh, Pittsburgh, PA, United States; 5 Departments of Neurology and Pathology, University of Pittsburgh Medical Center, Pittsburgh, PA, United States; 6 Independent Researcher, Pittsburgh, PA, United States; 7 Patient Author, Pittsburgh, PA, United States; 8 Division of Genetics and Genomic Medicine, Department of Pediatrics, UPMC Children’s Hospital of Pittsburgh, Pittsburgh, PA, United States; 9 Division of Pediatric Gastroenterology, Department of Pediatrics, UPMC Children’s Hospital of Pittsburgh, Pittsburgh, PA, United States; 10 Divison of Blood and Marrow Transplantation, Department of Pediatrics, UPMC Children’s Hospital of Pittsburgh, Pittsburgh, PA, United States; 11 Divisions of Pediatric Hematology and Oncology, and Palliative Medicine and Supportive Care, Department of Pediatrics, UPMC Children’s Hospital of Pittsburgh, Pittsburgh, PA, United States; 12 Division of Pediatric Hospital Medicine (Emeritus), Department of Pediatrics, UPMC Children’s Hospital of Pittsburgh, Pittsburgh, PA, United States

**Keywords:** autoimmune disease, hematopoietic stem cell transplantation, ITCH deficiency, lung disease, polymyositis, ubiquitin

## Abstract

**Background:**

Itchy E3 ubiquitin ligase deficiency (ITCH deficiency) is a rare monogenic immune dysregulation syndrome characterized by early-onset multisystem autoimmunity and significant morbidity and mortality. Only one prior case report has described a patient treated with hematopoietic stem cell transplantation (HSCT) with clinical improvement, and no established disease-modifying or curative therapy algorithm exists.

**Case description:**

We describe a female patient who presented in early childhood with chronic lung disease requiring tracheostomy and long-term mechanical ventilation and later developed progressive muscle weakness. She was diagnosed with steroid-responsive juvenile polymyositis and subsequently accumulated multiple autoimmune manifestations including autoimmune hepatitis, enteropathy, psoriasis, inflammatory arthritis, and Sjögren’s-like parotitis. Genetic testing ultimately identified compound heterozygous pathogenic variants in *ITCH*. Despite prolonged treatment with multiple immunosuppressive and biologic therapies, she remained prednisone dependent with poor disease control and substantial treatment-related morbidity. At 20 years of age, she underwent allogeneic hematopoietic stem cell transplantation. Following transplantation, she achieved sustained resolution of autoimmune disease activity, was successfully weaned from all immunosuppressive therapy, and was decannulated from chronic mechanical ventilation, with marked improvement in functional status and quality of life.

**Conclusions:**

This is the second reported case demonstrating the benefit of hematopoietic stem cell transplant (HSCT) in ITCH deficiency and provides evidence of durable multisystem clinical remission and recovery of long-standing pulmonary and musculoskeletal disease. These findings support consideration of HSCT as a therapeutic option in carefully selected patients with severe, refractory autoimmune manifestations due to ITCH deficiency and raise the possibility that earlier consideration in the disease course may mitigate cumulative disease- and treatment-related morbidity.

## 1 Introduction

In recent years, dysregulation of the ubiquitin system has emerged as a central mechanism underlying several monogenic inflammatory and autoimmune diseases, including otulipenia/otulin-related autoinflammatory syndrome, haploinsufficiency of A20, and linear ubiquitin chain assembly complex deficiency (1). Ubiquitination is a highly conserved post-translational process that regulates protein stability and signaling through targeted proteasomal degradation (2). This process is mediated by a multistep enzymatic cascade involving E1 activating enzymes, E2 conjugating enzymes, and E3 ubiquitin ligases, the latter of which confer substrate specificity. More than 600 E3 ubiquitin ligases are encoded in the human genome, underscoring their critical role in immune homeostasis (3).

The E3 ubiquitin ligase ITCH plays a key role in immune regulation through its involvement in T-cell receptor signaling, T-cell expansion, regulatory T-cell development, and B-cell maturation (4–6). Murine models deficient in ITCH demonstrate profound immune dysregulation characterized by impaired peripheral tolerance, excessive T-cell activation, elevated immunoglobulin levels, autoantibody production, and a skewed Th2 immune response (7). These findings highlight the importance of ITCH-mediated ubiquitination in maintaining immune balance.

In humans, biallelic pathogenic variants in *ITCH* cause Itchy E3 ubiquitin ligase deficiency (OMIM gene 606409), a rare autosomal recessive immune dysregulation syndrome characterized by early-onset multisystem autoimmunity (8, 9). Reported clinical manifestations include autoimmune hepatitis, enteropathy, thyroiditis, chronic lung disease, and characteristic facial dysmorphism (8). Management is largely supportive and organ-directed, and many patients experience progressive disease with substantial morbidity and mortality. Given the central role of ITCH in immune tolerance, replacement of the defective immune system through allogeneic hematopoietic stem cell transplantation (HSCT) has been proposed as a potential disease-modifying strategy in severe or refractory cases.

To date, only one patient with ITCH deficiency who has been successfully treated with HSCT has been reported in the literature (10). Here, we describe a patient with genetically confirmed ITCH deficiency and severe, longstanding multisystem autoimmune disease, including juvenile polymyositis, inflammatory arthritis, psoriasis, autoimmune enteropathy and hepatitis, and chronic interstitial lung disease requiring long-term tracheostomy and mechanical ventilation, who experienced marked and durable clinical improvement following HSCT despite nearly two decades of active disease and accrued organ damage. This case expands the clinical spectrum of ITCH deficiency and provides further evidence supporting HSCT as a potential therapeutic option in patients with severe immune dysregulation due to ITCH deficiency.

## 2 Case description

**Figure 1:**
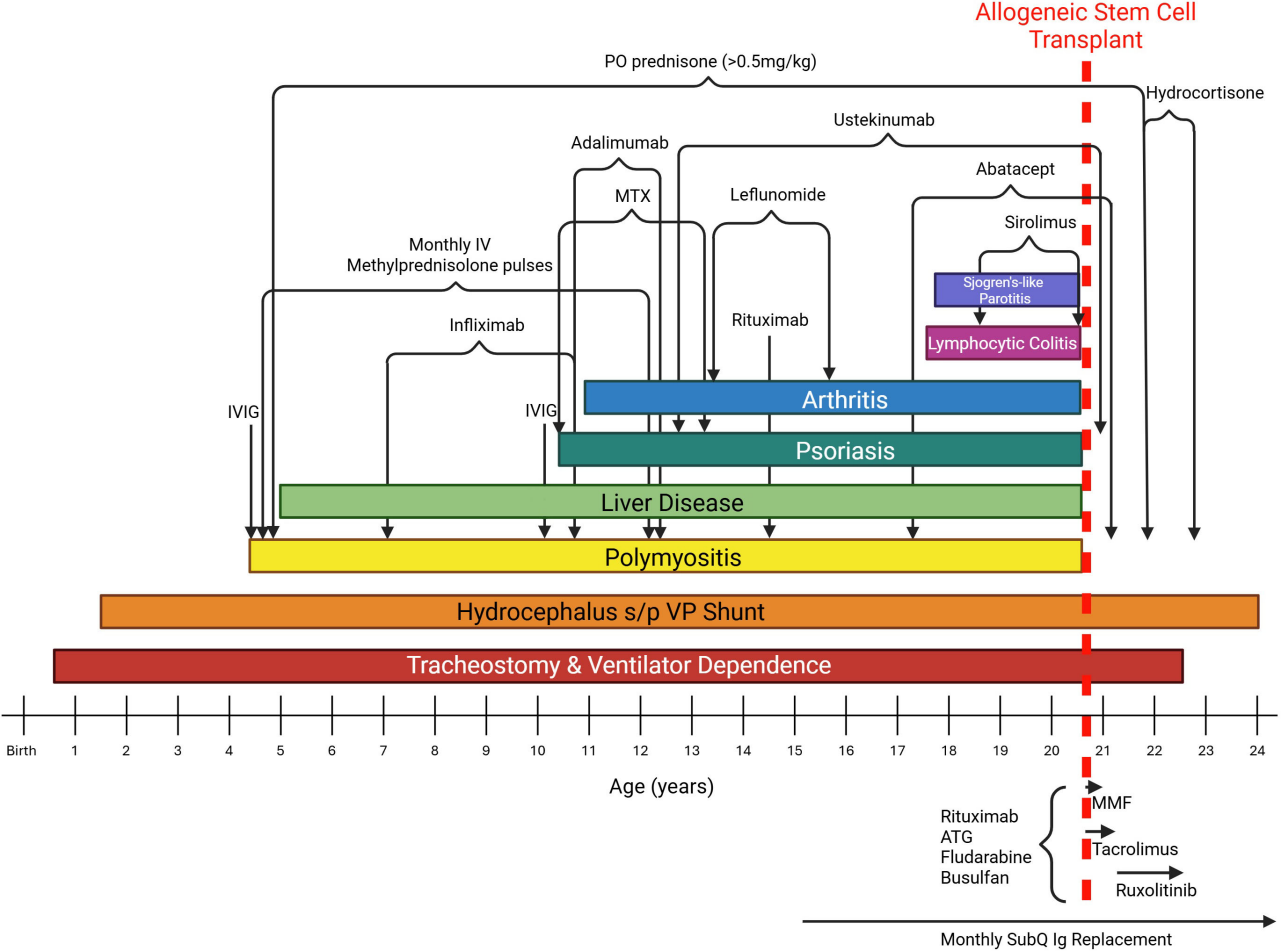
Timeline of disease manifestations, immunomodulatory therapies, and hematopoietic stem cell transplantation. The patient’s age (in years) is shown on the x-axis. Major clinical manifestations, including autoimmune disease features, are displayed as colored horizontal bars across above the timeline. Immunomodulatory and immunosuppressive therapies are indicated by horizontal black brackets corresponding to periods of treatment. Medications used for conditioning and immediate post–allogeneic hematopoietic stem cell transplantation are shown below the timeline. At last follow-up, the only ongoing immune-modulating therapy is subcutaneous immunoglobulin replacement. Abbreviations include: IVIG, intravenous immunoglobulin; MTX, methotrexate; SubQ Ig, subcutaneous immunoglobulin.

The patient was born full term to non-consanguineous parents, with a prenatal course complicated by intrauterine growth restriction. At seven weeks of age, she required intubation for respiratory syncytial virus bronchiolitis and subsequently experienced recurrent hospitalizations for respiratory failure and failure to thrive. She underwent tracheostomy with long-term mechanical ventilation and gastrostomy tube placement by seven months of age, followed by ventriculoperitoneal shunt placement for symptomatic hydrocephalus at 18 months of age. Evaluation by the genetics team for skeletal dysplasia due to macrocephaly, bilateral hip dysplasia, and growth deficiency during infancy did not reveal a unifying diagnosis at that time (**Figure 1**).

She remained clinically stable until four years of age, when she developed bilateral ptosis, progressive lower extremity weakness, and frequent falls. Initial neurologic evaluation, including brain and spine magnetic resonance imaging was unremarkable. Cerebrospinal fluid analysis demonstrated elevated protein at 94 mg/dL (reference range 12–60 mg/dL) with minimal pleocytosis. She was empirically treated with intravenous immunoglobulin for presumed Miller–Fisher variant of Guillain–Barré syndrome, with transient improvement followed by symptom recurrence.

**Figure 2:**
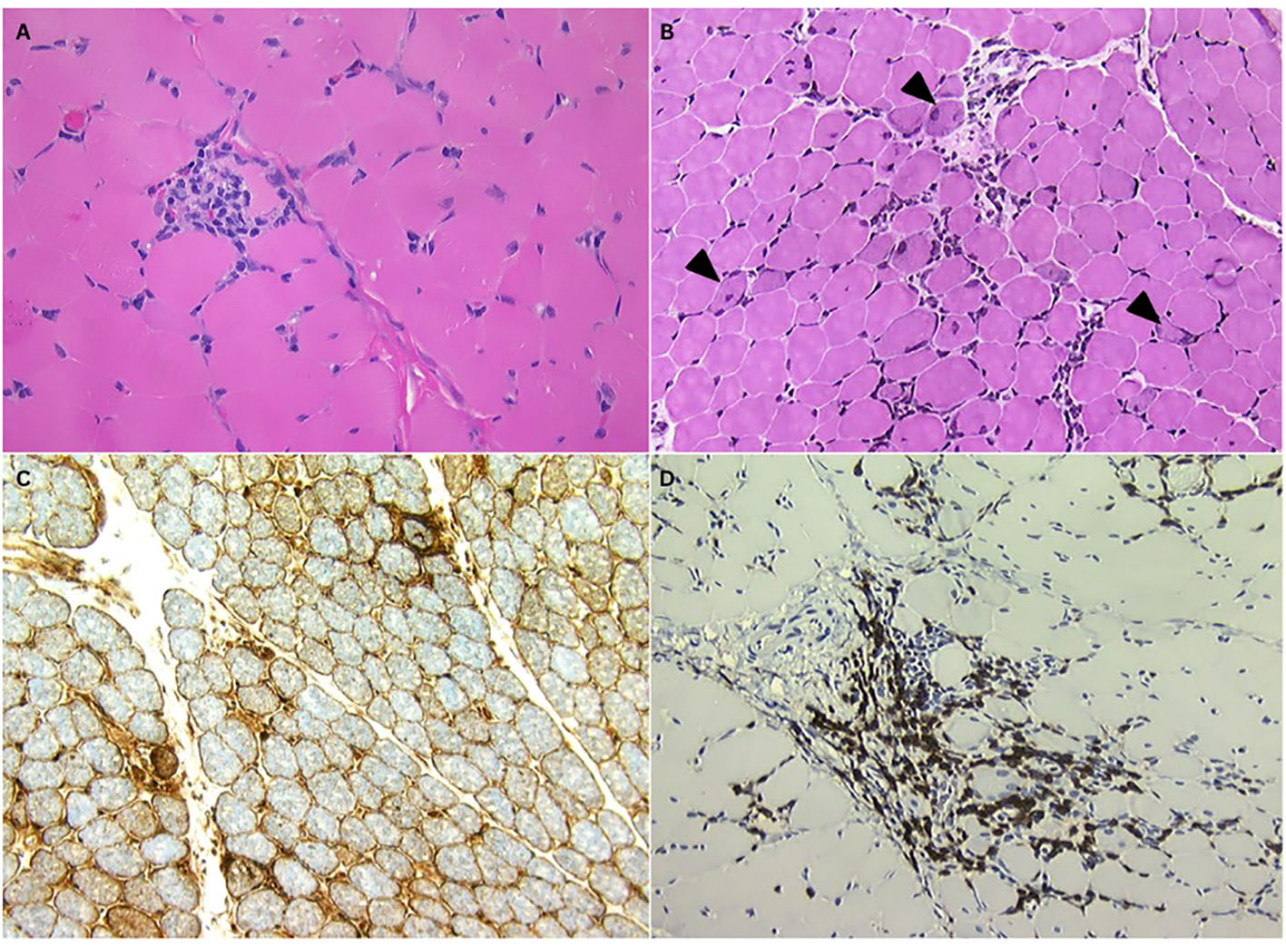
Open muscle biopsy of right quadriceps muscle. **(A)** H&E, paraffin section: Myofiber invasion by mononuclear cells, **(B)** H&E, frozen section: Mononuclear inflammatory cells in the endomysium and around a perimysial blood vessel. There are scattered regenerating myofibers (arrowheads), **(C)** MHC1 immunostain, frozen section: Diffuse abnormal sarcolemmal and some sarcoplasmic upregulation of MHC Complex Class I, **(D)** CD3+ immunostain, frozen section: The majority of inflammatory cells are noted to be CD3+ T cells.

On readmission for progressive weakness, serum creatine phosphokinase was markedly elevated at 4,400 IU/L (reference range 30–150 IU/L). Magnetic resonance imaging of the pelvic girdle revealed diffuse bilateral muscle edema and enhancement, consistent with an inflammatory myopathy. In the absence of characteristic cutaneous findings, juvenile dermatomyositis was considered less likely. Electromyography demonstrated a myopathic pattern, and muscle biopsy revealed numerous degenerating and regenerating fibers with multifocal endomysial and perivascular inflammatory infiltrates and focal myofiber invasion, most consistent with polymyositis (**Figure 2**). Special stains excluded mitochondrial, lipid, glycogen, and amyloid pathology, and immunohistochemistry for membrane attack complex was negative in endomysial capillaries.

Rheumatology consultation confirmed persistently elevated CPK, aldolase, and transaminases, with preserved renal function and normal electrolytes. She was treated with high-dose intravenous methylprednisolone (30 mg/kg daily for three days), resulting in a robust clinical and biochemical response, with creatine phosphokinase declining from 9,000 IU/L to 200 IU/L and aldolase from 200 U/L to 10 U/L. However, attempts to taper corticosteroids led to recurrent weakness and laboratory relapse, necessitating ongoing pulse intravenous steroid therapy. Myositis-specific antibodies and extractable nuclear antigen testing were negative.

By five years of age, she developed persistent transaminase elevations (ranging from 400-1600) not attributable to muscle disease (**Figure 1**). Liver biopsy demonstrated centrilobular hepatitis. Extensive metabolic and genetic evaluations revealed deficiencies in respiratory chain complexes II, I–III, and II–III, though these findings did not fully account for her multisystem phenotype.

Over subsequent years, multiple steroid-sparing and biologic therapies were trialed without durable disease control. Tumor necrosis factor inhibition (Infliximab and Adalimumab), methotrexate, intravenous immunoglobulin, IL-12/23 inhibition (Ustekinumab), and B-cell–directed therapy (Rituximab) produced limited or organ-specific responses and failed to permit sustained corticosteroid tapering (**Figure 1**). Rituximab therapy was further complicated by a severe infusion reaction and hypogammaglobulinemia requiring ongoing immunoglobulin replacement. During this period, she developed additional autoimmune manifestations, including psoriasis, inflammatory arthritis requiring repeated intra-articular steroid injections, autoimmune enteropathy, and progressive pulmonary disease. Chest computed tomography demonstrated bronchial wall thickening, bronchiectasis, and mosaic attenuation, consistent with evolving interstitial lung disease. A lung biopsy obtained via video-assisted thoracoscopic surgery (VATS) demonstrated predominantly emphysematous changes, which were felt to be secondary to long-term mechanical ventilation. There were focal areas of chronic inflammatory infiltrate associated with intraluminal macrophages, including cells with foamy cytoplasm; however, overall inflammatory changes were minimal. Patchy areas of mild fibrosis were also present, as evidenced by elastic trichrome staining, but without significant fibrosis remodeling. In addition to intrinsic lung pathology, pulmonary function was further compromised by profound muscle weakness related to polymyositis and severe deconditioning.

At 13 years of age, whole exome sequencing identified a maternally-inherited heterozygous pathogenic *ITCH* variant (c.599dupC; p.Ser201Ilefs*8). Deletion/Duplication studies revealed a second, *de novo* exons 25 and 26 deletion on the alternate allele, confirming a compound heterozygous state consistent with autosomal recessive ITCH deficiency (9). Despite escalating combination immunosuppressive regimens, she remained prednisone dependent, and attempts to taper corticosteroids below 0.5 mg/kg/day precipitated flares of myositis, pulmonary disease, or cutaneous inflammation. Over time, she accrued substantial treatment-related morbidity, including recurrent serious infections (bacterial pneumonias, tracheitis, cellulitis, episodes of sepsis), osteopenia with pathologic fractures, steroid-induced hypertension, and Cushingoid features. Additional clinical and laboratory findings regarding this patient’s initial clinical course have been published previously (9).

In late adolescence, additional autoimmune manifestations emerged, including lymphocytic colitis and Sjögren’s-like parotitis, both supported by tissue diagnosis. Sirolimus was added to her immunosuppressive medication without much clinical impact. Given progressive, multisystem immune dysregulation refractory to numerous immunosuppressive and biologic therapies, hematopoietic stem cell transplantation (HSCT) was increasingly considered. However, her complex medical status, including multiple active organ system involvements, chronic infections, and profound steroid-related morbidity, raised significant concern about her ability to tolerate transplant-related toxicity. Psychosocial barriers further complicated the decision: being intensely medicalized since infancy, the patient initially regarded HSCT, first proposed at 18 years of age, as just another high-risk intervention of uncertain value, whereas her mother, a nurse versed in the natural history of ITCH deficiency and the patients cumulative complications (her medication list included 50 different medications and she was ventilator dependent 24 hours per day), recognized that without HSCT the prognosis was bleak. Following prolonged multidisciplinary counseling and shared decision-making, she consented to allogeneic HSCT at 20 years of age with the hope that it could lead to steroid-independence and improved quality-of-life.

The patient received hematopoietic stem cells from a 12/12 HLA-matched unrelated bone marrow donor. Conditioning included busulfan administered on days −7 to −4 (target AUC 60 mg·h/L), rabbit anti-thymocyte globulin on days −7 to −5 (3 mg/kg/dose), and fludarabine at 40 mg/m²/day on days −5 to −2. Graft-versus-host disease prophylaxis consisted of abatacept, tacrolimus, and mycophenolate mofetil, with rituximab administered for post-transplant lymphoproliferative disorder prophylaxis. She achieved neutrophil engraftment on Day +11; platelet engraftment on Day +18.

Early chimerism studies demonstrated robust donor engraftment in the lymphoid and myeloid compartments, but T cell chimerism of <50%. She therefore received donor lymphocyte infusions to boost this compartment at day +23 (1.14 x 10^6 CD3 cells/kg infused) and again on day +134 post-transplant (5.70 x10^5 CD3 cells/kg infused). (**Table 1**). She did not experience any infectious complications nor graft-versus-host disease in the immediate post-transplant period.

**Table 1:** Immune reconstitution* parameters before transplant and at 3 months, 1 year, 2 years, and 3 years post-transplant.

	Reference range**	Pre-HSCT	3 months post-HSCT	1 year post-HSCT	2 years post-HSCT	3 years post-HSCT
CD3+ (cells/µL)	856-2669	888	174	358	1107	1065
CD4+ (cells/µL)	491-1734	386	70	209	647	642
CD8+ (cells/µL)	162-1074	485	101	141	445	402
CD4+/CD45RA+ (cells/µL)	41-1121	77	22	63	323	321
CD4+/CD45RA+/CD62L+ (cells/µL)	152-1127	66	22	71	317	327
CD19+ (cells/µL)	73-562	107	0	198	542	584
CD19+/CD27+/IgD- (cells/µL)	1.5-31	0.5	–	–	–	2.2
NK cells (cells/µL)	108-680	430	105	92	348	227
IgG (cells/µL)	610-1620	881	769	987	950	971
IgA (cells/µL)	84-500	<2	<2	<2	6	67
IgM (cells/µL)	35-242	35	18	34	34	47
Chimerism: PB MC	n/a	–	95%	90%	86%	97%
Chimerism^¥^:T	n/a	–	34%	73%	91%	92%
Chimerism^¥^:B	n/a	–	94%	90%	90%	91%
Chimerism^¥^:M	n/a	–	95%	90%	85%	89%

*Immune reconstitution was assessed longitudinally following hematopoietic stem cell transplantation. Sustained full donor engraftment was observed in myeloid and B-cell compartments, with gradual normalization of T-cell counts over time.

** Age-adjusted reference range at the time of testing.

^¥^Chimerism percentages represent donor-derived cells assessed in peripheral blood mononuclear cells (PBMCs) and lineage-specific compartments (T cells, B cells, and myeloid cells).

**Figure 3:**
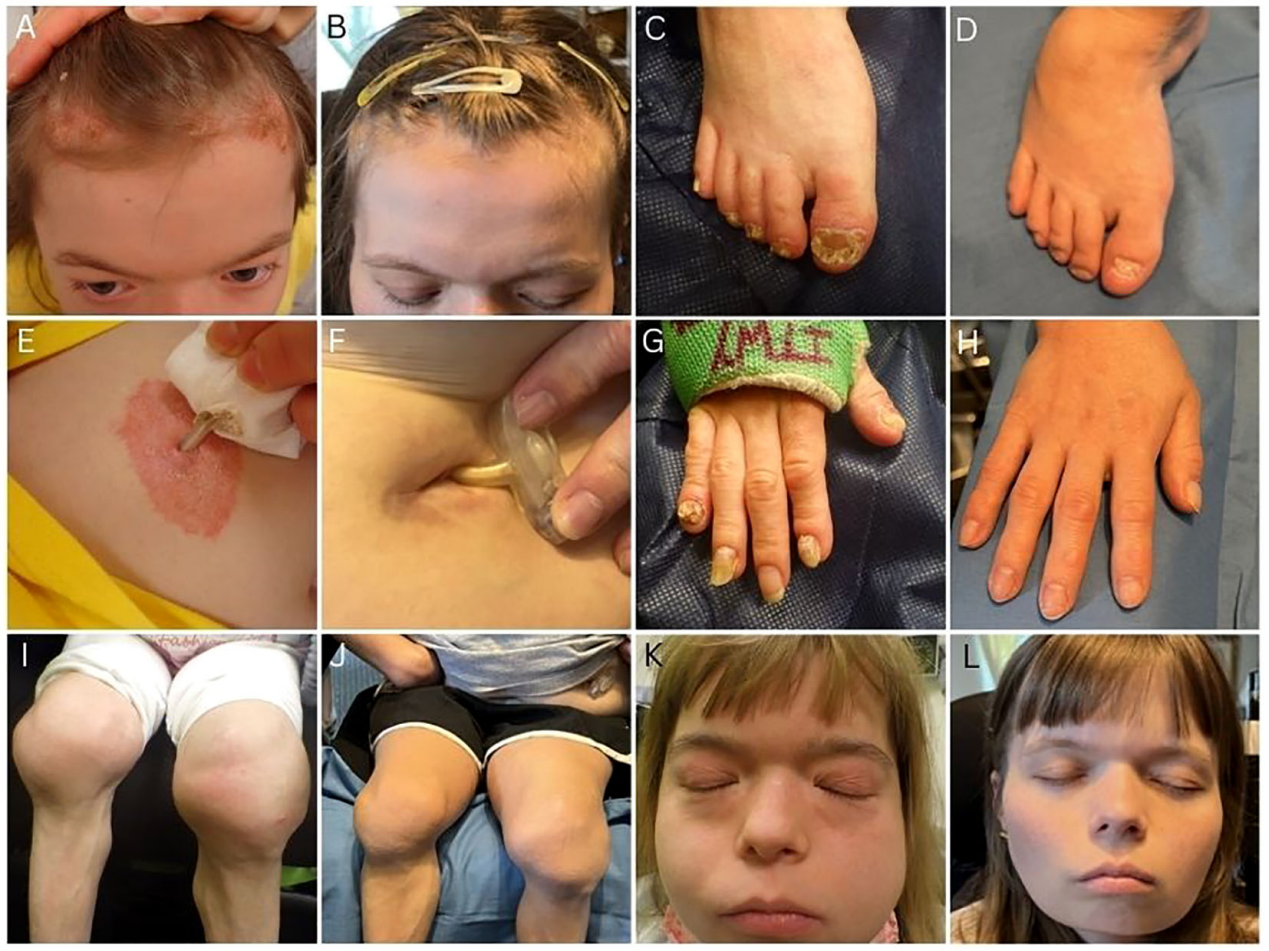
Clinical images pre- and post-transplant. *Pre-transplant:* **(A)** Scalp psoriasis, **(C)** toenail psoriasis, **(E)** psoriasis around gastrostomy tube site, **(G)** fingernail psoriasis, **(I)** inflammatory arthritis of the knee, **(K)** psoriasis on face, cushingoid appearance, and parotitis. *36 months post-transplant:* **(B)** clear scalp, **(D)** clear toenails, **(F)** clear gastrostomy tube site, **(H)** clear fingernails, **(J)** resolution of arthritis, **(L)** resolution of cushingoid appearance and parotitis.

Three months post-transplant, she developed a pericardial effusion attributed to tacrolimus, which resolved following transition to ruxolitinib. She also continued to demonstrate mixed chimerism of her T-cells (34%). Despite this, she had a remarkably uncomplicated transplant course with progressive, multisystem improvement. Immunosuppressive therapies, including biologics (ustekinumab and abatacept) and corticosteroids, were successfully tapered (**Figure 1**). Psoriatic skin disease resolved (**Figure 3**), inflammatory arthritis entered remission without the need for intra-articular injections that had previously been required every 3–6 months for symptom control, and pulmonary function improved with gradual reduction in ventilator dependence, transitioning from 24-hour dependence to intermittent “sprints” by 7 months post-transplant.

By one year post-transplant, she required only physiologic hydrocortisone replacement and ruxolitinib for graft-versus-host prophylaxis. At 18 months post-transplant, she no longer required ventilatory support at all and was successfully decannulated after lifelong tracheostomy dependence. By two years post-transplant, all immunosuppressive therapy, including the ruxolitinib, had been discontinued without evidence of autoimmune disease recurrence or chronic graft-versus-host disease. Sustained full donor engraftment was observed in myeloid and B-cell compartments, with gradual normalization of T-cell counts (**Table 1**).

Now three years post-transplant, she remains free of active autoimmune disease off all immunosuppression for over one year, and has experienced no major infections, pulmonary exacerbations, or fractures. She no longer requires ventilatory or inhaled respiratory support (she was on at least 4 nebulizer treatments and pulmonary toilet therapy pre-transplant). She continues immunoglobulin replacement for persistent hypogammaglobulinemia but has otherwise demonstrated marked improvement in functional independence and quality of life.

## 3 Discussion

Several cases of ITCH deficiency have been reported to date, including those described previously (8–12). Across reported cohorts, patients commonly present with dysmorphic facial features, failure to thrive, macrocephaly, chronic lung disease, and more than half develop autoimmune manifestations such as hepatitis, enteropathy, hypothyroidism, and type 1 diabetes mellitus (10). Disease course is often severe, with significant morbidity and reported mortality, most frequently related to progressive pulmonary involvement. To date, only one patient with ITCH deficiency has been reported to undergo hematopoietic stem cell transplantation (HSCT) with sustained clinical benefit (10).

In the previously reported HSCT case, the patient presented with arthritis and dysmorphic features and subsequently developed psoriasis, uveitis, inflammatory bowel disease, diabetes, and growth failure. Following HLA-matched sibling transplantation, that patient remained off immunosuppressive therapy at one and two years post-transplant, with marked multisystem improvement and catch-up linear growth (10). While encouraging, that report did not include patients with longstanding ventilator-dependent pulmonary disease.

Our patient expands the clinical spectrum of ITCH deficiency in several important ways. She appears to be the first reported case with severe, early-onset inflammatory myopathy consistent with polymyositis, a condition that is rare in pediatric populations (13). Her myositis was initially steroid responsive but proved refractory to multiple biologic and disease-modifying agents, mirroring treatment challenges observed in adult polymyositis (14). She also developed inflammatory arthritis despite therapy with anti-TNF agents and methotrexate, which are typically effective first-line treatments for juvenile idiopathic arthritis (15). Other autoimmune manifestations, including psoriasis and enteropathy, similarly demonstrated incomplete or transient responses to conventional immunomodulatory strategies.

Pulmonary disease represented one of the most clinically significant aspects of her course. Chronic lung involvement is common in ITCH deficiency, with approximately 90% of patients in the original Amish cohort demonstrating pulmonary disease and several deaths attributed to respiratory failure (8). In contrast to previously reported patients, our patient required continuous mechanical ventilation from infancy, reflecting both disease severity and early onset. Remarkably, despite nearly two decades of chronic lung disease with some evidence of fibrosis, HSCT was associated with progressive pulmonary recovery, culminating in successful decannulation approximately 18 months post-transplant. This degree of reversibility suggests that immune-mediated mechanisms may contribute substantially to pulmonary pathology in ITCH deficiency, even in advanced disease.

Beyond organ-specific improvement, the patient has demonstrated meaningful and sustained functional recovery. Years of corticosteroid exposure resulted in severe osteoporosis with pathologic fractures and severe scoliosis, and chronic inflammatory myopathy led to profound muscle weakness and deconditioning. At the time of HSCT, she was wheelchair-dependent, able to stand independently for only a few seconds, and unable to walk beyond just a few steps with a walker. Following immune reconstitution, she has shown steady gains in muscle strength and endurance through intensive physical and occupational therapy. She is now able to stand independently for several minutes and ambulate distances exceeding 400 feet with a walker. Three years after transplantation, she was able to walk down the aisle with a decorated walker as a bridesmaid at her cousin’s wedding, reflecting substantial functional recovery and improved quality of life.

The patient had also received growth hormone therapy for documented growth deficiency affecting stature, skeletal development, and muscle mass, with daily injections administered from 2004 through 2021. Growth hormone was discontinued prior to transplantation, and she remains off therapy, with gradual improvements in muscle mass continuing through rehabilitation and physical therapy.

At three years post-transplant, the patient remains off all immunosuppressive therapy with no evidence of autoimmune disease recurrence and sustained improvement in pulmonary, musculoskeletal, and functional outcomes. Importantly, HSCT was undertaken after years of refractory disease and accrued organ damage, underscoring the potential for meaningful benefit even when transplantation is performed later in the disease course. Nevertheless, this report is limited by its single-patient design, and the long-term durability of immune reconstitution and organ recovery in ITCH deficiency remains unknown.

In summary, this case provides further evidence that HSCT can induce durable multisystem remission in patients with severe ITCH deficiency and highlights the potential for recovery of advanced pulmonary and musculoskeletal disease. While experience remains limited, with only two reported cases of successful HSCT in ITCH deficiency to date, both patients demonstrated substantial and sustained clinical improvement following immune reconstitution. Notably, review of published cases suggests that individuals with ITCH deficiency uniformly develop significant clinical disease rather than remaining asymptomatic, supporting the concept that this disorder follows a progressive course once immune dysregulation becomes established.

Although definitive recommendations regarding timing cannot be made based on the current evidence, this case raises the possibility that consideration of HSCT earlier in the disease trajectory, after diagnosis and initial observation, but prior to the development of severe refractory disease, may reduce cumulative organ damage and treatment-related morbidity. In our patient, prolonged reliance on corticosteroids and other immunosuppressive therapies was associated with substantial iatrogenic complications, including severe osteopenia with pathologic fractures, cataracts, growth restriction, and long-term functional impairment. Earlier immune reconstitution may have mitigated some of these sequelae. Future multicenter experience will be essential to better define the natural history of ITCH deficiency and to clarify the optimal timing of HSCT in affected patients.

## 4 Patient perspective

The patient describes herself as a “mystery wrapped in a riddle.” Her mother recalls that from infancy, the patient was frequently in and out of the hospital for admissions and extensive testing, which she describes as “overwhelming.” Both the patient and her mother identify tracheostomy dependence as the most challenging aspect of her illness, noting that it altered holiday plans, limited travel, required around-the-clock nursing care, and demanded significant time for frequent respiratory treatments. Despite these challenges, the family made it a priority to provide the patient with a “normal life” including attending school, participating in Girl Scouts, and visiting local amusement parks, where she enjoyed riding roller coasters.

Her mother describes receiving the genetic diagnosis at 13 years of age as “very relieving,” explaining that “now we know what it is, now you have an answer, even if it’s very rare.” Despite having a diagnosis, it was difficult to witness the patient’s decline during her late teens and twenties as she became progressively weaker and appeared to accumulate diagnoses more rapidly, describing a “cascade” of complications. For example, the patient was unable to attend school for most of her sophomore year (2017–2018) after undergoing a scheduled hip osteotomy secondary to hip dysplasia and flattened femoral head causing significant daily pain. This was followed by kidney stones and pyelonephritis requiring vasopressor support in the intensive care unit, multiple stent placements and surgical procedures, and subsequent hospital-acquired pneumonia.

The parents describe that when the idea of a bone marrow transplant was first proposed, they felt excited, while the patient herself had mixed feelings and was initially fearful. With progressively declining quality-of-life, the patient ultimately decided that the potential benefits of steroid independence outweighed the risks of HSCT. She described a clear turning point when her small dog jumped onto her lap and she sustained a femoral fracture, reinforcing how fragile her health had become and that her quality of life was so poor that she had to do “something.” She reflected that even if the only outcome of transplantation were the ability to discontinue steroids, it would be “worth it.”

After a year of delays related to the COVID-19 pandemic, the family reports that the transplant process went as smoothly as possible and that, over time, the patient gradually regained function. Both the patient and her family would recommend discussing bone marrow transplantation earlier in the disease course, before the accumulation of a lifetime of complications. When asked about the most significant change following transplantation, both the patient and her parents described “everything,” while specifically highlighting that decannulation provided the family with substantially greater freedom. The patient’s current goals include obtaining her driver’s license, learning to swim, artistically painting her nails again, attending college, and securing employment.

## Data Availability

The original contributions presented in the study are included in the article/supplementary material. Further inquiries can be directed to the corresponding author.
